# Editorial: Analyzing male reproductive risk, understanding molecular targets, and developing treatments

**DOI:** 10.3389/fendo.2024.1439809

**Published:** 2024-06-27

**Authors:** Xiang Xiao, Huitao Li, Cibele S. Borges, C. Yan Cheng

**Affiliations:** ^1^ Center for Reproductive Health, School of Pharmaceutical Sciences, Hangzhou Medical College (Zhejiang Academy of Medical Sciences), Hangzhou, China; ^2^ Zhejiang Engineering Research Center for Innovation and Application of Intelligent Radiotherapy Technology, and Department of Anesthesiology and Perioperative Medicine, The Second Affiliated Hospital of Wenzhou Medical University, Wenzhou, China; ^3^ Key Laboratory of Environment and Male Reproductive Medicine of Wenzhou, Wenzhou, China; ^4^ Laboratory of Tissue Biology and Developmental Toxicology, Department of Bioscience - CCBS, UFERSA - Federal Rural University of Semi-Arid Region, Mossoró, Brazil; ^5^ Department of Urology and Andrology, Sir Run-Run Shaw Hospital, Zhejiang University School of Medicine, Hangzhou, China

**Keywords:** male fertility, sperm, testis, semen quality, endocrine disruption

## 1 Introduction

Male fertility is profoundly impacted by environmental factors from lifestyle, diseases, and toxicants. However, the specific molecular mechanisms of these external influences on male reproductive health remain unclear. This Research Topic aimed to gain insights into how elements like toxicants, diseases, medications, and nutrition affect male fertility at the cellular and molecular levels. Better characterizing pathways linking diverse external stimuli to outcomes could assist in improving diagnostic and therapeutic approaches for infertility. Identifying shared regulatory mechanisms across conditions may highlight targets for non-invasive strategies or restoring function. Ultimately, a deeper understanding of resilience and vulnerability could accelerate enhancing diagnosis and treatment impacted by environment and health. This editorial provides an overview of key contributions advancing the understanding of male fertility regulation against stressors.

## 2 Spermatogenesis processes and regulation

Several studies featured in this Research Topic provided important new understandings of the dynamic cellular processes governing normal spermatogenesis and their disruption. Xiao et al. revealed a novel role for the circulating signaling molecule sICAM-1 in regulating the integrity of the blood-testis barrier and adhesion between Sertoli cells and germ cells through inhibition of SRC family kinase signaling pathways in Sertoli cells. By downregulating SRC activity, sICAM-1 was shown to facilitate the essential transport of germ cells through the seminiferous epithelium required for sperm production.

Li et al. reported the intriguing finding of positive trends over time in specific sperm motility parameters like velocity, even as more conventional measures of semen quality like count declined in their analysis of over 49,189 semen samples. Sperm motility is a highly heritable trait that could reflect adaptive responses to environmental pressures. This study points to the possibility of compensatory mechanisms offsetting certain impairments and warrants further exploration of factors modulating this critical fertility trait. Together, these articles provided novel mechanistic insights into the dynamic cellular adhesion events and regulatory pathways that govern normal human spermatogenesis.

## 3 Disease states and lifestyle impacts

Several studies investigated the effects of specific disease conditions and short-term lifestyle influences on male reproductive health at the molecular level. Osadchuk et al. provided a comprehensive analysis linking cigarette smoking to multi-level impairments in semen quality indicators and general male health through disrupted zinc homeostasis, oxidative damage, metabolic dysregulation, and inflammation within a large Russian population. This work demonstrated ethnicity-dependent sensitivities to smoking toxicity.

Liu et al. associated fluctuations in Leydig cell lipid metabolism and related hormone levels with the ability to retrieve focal sperm samples from patients of different ages with Klinefelter syndrome, indicating potential therapeutic targets related to age. Sun et al. identified progressive transcriptional changes driving impaired spermatogenesis in undescended testes from cryptorchidism patients through RNA sequencing and bioinformatics analyses.

Additionally, Falvo et al. demonstrated how even a short five-week high-fat diet period impaired rat testicular functions through disruptions to mitochondria, antioxidant defenses, barrier integrity, and signaling cascade. These studies provided novel disease- and lifestyle-specific insights into pathological influencers of male reproductive health.

## 4 Connections to broader health

Several contributions examined relationships between male fertility and broader indicators of physiological wellness. Huang et al. analyzed data from over 3,625 American males, finding that visceral adiposity assessed through a novel metric strongly predicted the prevalence of erectile dysfunction. This highlights the clinical utility of analyzing body fat distribution patterns for sexual health risk evaluation.

Additionally, Simón et al. reviewed evidence that various bioactive compounds commonly found in plants may help mitigate potential infertility risks associated with cancer therapies by protecting against oxidative stress, inflammation, and other damaging effects in testicular cells through diverse protective mechanisms revealed in animal and cellular research. Evaluating natural agents as alternative or adjuvant options represents an impactful area for future therapeutic development. Together, these studies delineated links between specific health parameters and male reproductive function.

## 5 Toxicant exposure propagation

Lee et al. provided a clear example of how environmental toxicity can propagate impairment from one organ system to negatively impact male fertility through the disruption of metabolic crosstalk. Their study linked exposure to the ubiquitous contaminant perfluorooctane sulfonate (PFOS) to perturbed hepatic lipid metabolism and related gene expression in mice. This was shown to subsequently influence testicular structure and function through increases in fatty acid metabolites and perturbation of testicular lipid pathways.

The findings showed that PFOS exposure impairs male reproductive health by disrupting the normal balance of fatty acid metabolism between the liver and testes. This work exemplified how elucidating causative toxicological pathways can reveal promising molecular targets for monitoring or mitigating contamination-related health effects, here pointing to lipid regulatory networks. It highlighted the utility of investigating connectivity between organ toxicodynamics to fully characterize toxicity mechanisms.

## 6 Significant advances and conclusions

Collectively, the diverse studies on this topic significantly advanced the understanding of male fertility as resilient yet vulnerable to various stressors. Common mechanisms and molecular signatures were identified across studies, providing opportunities for future research. Novel insights were provided into dynamic processes like cellular junctions, motility, and transcriptional profiles, as well as tissue crosstalk and lifestyle influences on fertility regulation.

Outstanding questions warranting further exploration were also highlighted, including compensatory responses, ethnicity influences, and optimizing targeted therapies. Achieving deeper insights into interconnected regulatory pathways influencing fertility across conditions will guide development of improved diagnostics, personalized infertility treatment strategies, and prevention methods.

Continued multidisciplinary efforts to map the systemic complexities of fertility regulation and identify intervention points at multiple scales will further the overarching goal of enhancing global reproductive health. This Research Topic meaningfully advances that aim through advancing knowledge of spermatogenesis, disease pathogenesis, toxic impacts, and links between overall health and fertility outcomes. Overall, the work here collectively pushes boundaries in understanding male reproductive resilience and vulnerabilities.

## Author contributions

XX: Conceptualization, Writing – original draft, Writing – review & editing. HL: Writing – review & editing. CB: Writing – review & editing. CC: Writing – review & editing.

